# Donor genetic backgrounds contribute to the functional heterogeneity of stem cells and clinical outcomes

**DOI:** 10.1002/sctm.20-0155

**Published:** 2020-08-24

**Authors:** Ting Wang, Juan Zhang, Jinqi Liao, Fan Zhang, Guangqian Zhou

**Affiliations:** ^1^ Department of Medical Cell Biology and Genetics, Guangdong Key Laboratory of Genomic Stability and Disease Prevention, Shenzhen Key Laboratory of Anti‐Aging and Regenerative Medicine, and Shenzhen Engineering Laboratory of Regenerative Technologies for Orthopaedic Diseases, Health Science Center Shenzhen University Shenzhen People's Republic of China; ^2^ Guangdong Key Laboratory for Biomedical Measurements and Ultrasound Imaging School of Biomedical Engineering, Shenzhen University Health Science Center Shenzhen People's Republic of China; ^3^ Lungene Scientific Ltd. Shenzhen People's Republic of China; ^4^ Department of Endocrinology and Metabolic Diseases Peking University Shenzhen Hospital, Shenzhen Peking University‐The Hong Kong University of Science and Technology Medical Center Shenzhen People's Republic of China

**Keywords:** cellular therapy, clinical translation, genomics, hematopoietic stem cells (HSCs), hematopoietic stem cell transplantation, induced pluripotent stem cells (iPSCs), mesenchymal stem cells (MSCs), stem cells

## Abstract

Stable and sustainable stem cell sources for stem cell‐based therapies are scarce and a key bottleneck for clinical applications. The regenerative potential of stem cells is usually attributed to several allogeneic or even autologous donor‐related factors. Genetic background and epigenetic variations in different individuals may significantly affect the functional heterogeneity of stem cells. Particularly, single‐nucleotide polymorphisms (SNPs) have been implicated in diseases with monogenetic or multifactorial and complex genetic etiologies. However, the possible effects of individual SNPs on donor stem cells remain far from fully elucidated. In this Perspective, we will discuss the roles played by donor genetic traits in the functional heterogeneity of induced pluripotent stem cells, mesenchymal stem cells, and hematopoietic stem cells and their implications for regenerative medicine and therapy.

## Graphical abstract

Donor genetic background contributes to stem cell functional heterogeneity: any formal clarification and recommendation with regard to the impact of single‐nucleotide polymorphisms carried with stem cell on the outcome of stem cell therapy will eventually require investigations in patients with respect to the design of clinical trials.

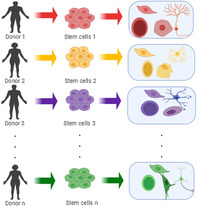

**Significance statement**

In the past decades, thousands of genetic variations termed single‐nucleotide polymorphisms (SNPs) have been identified, many of which are likely associated with complex human diseases that were previously hypothesized to have other unique genetic drivers. Genetic studies are rapidly being extended to stem cell research and regenerative medicine models. Considering the impact of SNPs in the etiology of diseases, it is reasonable to consider that stem cells carrying disease‐associated SNPs should not be transplanted onto the recipients with the same disease.

## 1 INTRODUCTION

Rapid advances in stem cell research have brought revolutionary progress to modern biology and medicine. These include the understanding of stem cell function, tissue development and growth, maintenance of tissue homeostasis, tissue regeneration, aging/degeneration, and therapies for diseases that cannot be cured with conventional medicine.^1^, ^2^ As of March 2020, the number of stem cell‐based registered clinical trials (www.ClinicalTrials.gov) has exceeded 5000, which is greater than any other single therapeutic technique or approach. As expected, the majority of these trials use hematopoietic stem cells (HSCs) and mesenchymal stem cells (MSCs) that can be acquired from various tissue sources.

Several major stem cell types, including ESCs, induced pluripotent stem cells (iPSCs), adult stem cells, and stem cells harvested from the fetus and related tissues (eg, umbilical cord blood, Wharton jelly from the umbilical cord, amnion, and placenta), have been comprehensively described and are being studied or used in the clinic.^3^ Whereas ESCs and iPSCs display pluripotent differentiation capabilities, most adult stem cell types, such as HSCs and MSCs derived from the bone marrow or other sources, have limited differentiation potential. Stem cells used in the clinic can be either allogeneic or autologous, depending on donor or tissue source availability. All stem cell types have relative advantages and limitations, and no single cell type meets all the optimal criteria for clinical applications.^4^ Thus, a wide variety of parameters should be considered for optimizing therapeutic efficacy while minimizing the potential for serious adverse events. This is particularly important in light of the goal of precision medicine; an initiative and effort that aims to make advances in tailoring medical care to the individual on a genetic and molecular basis.^5^

It is generally thought that major stem cell functions are induced by functional and/or architectural replacement of damaged tissue, paracrine signaling, or cellular mechanisms supporting self‐restoration of the diseased tissue or organ. Remarkably, these mechanisms are poorly defined for stem cell‐based therapy. In these cases, the mode‐of‐action of stem cells at the genetic and molecular levels should be elaborated for an in‐depth understanding of therapeutic mechanisms. Additionally, stem cell functionality and therapeutic efficacy may be influenced by several donor‐originated factors such as age, metabolic status, disease condition, genotype, and disease susceptibility‐related genetic background.^6^, ^7^, ^8^, ^9^

Evidence supporting the importance of donor genetic backgrounds underlying stem cell functional heterogeneity is emerging. In this Perspective, we summarize the current research and discuss the potential contribution of genetic backgrounds in order to advance the field of stem cell‐based therapy.

## 2 GENETIC BACKGROUNDS DRIVE iPSC FUNCTIONAL HETEROGENEITY

Emerging data have demonstrated that human iPSC technology has tremendous potential as cellular models for human diseases. However, variable genetic and phenotypic traits could restrict human iPSC applications. One previous study reported that 25 human iPSC lines were constructed from the same set of somatic tissues derived from different individuals.^10^ Single‐cell RNA‐sequencing (RNA‐seq) was performed for each cell line and resultant data were evaluated to identify the main source of heterogeneity. These comprehensive experiments indicated that genetic variations between different donors were the primary source of unique transcriptional differences between lines and that genetic background differences accounted for the majority of human iPSC functional heterogeneity. Additionally, genome‐wide profiling was carried out on 711 human iPSC lines derived from 301 healthy donors by the Human Induced Pluripotent Stem Cells Initiative.^11^ These data suggested that 5% to 46% of the variations in different human iPSC phenotypes, including in the epigenome, transcriptome, proteome, cell differentiation capacity, and cellular morphology, stemmed from genetic differences between individuals. Furthermore, RNA‐seq analysis and linear mixed models identified the genetic origins affecting gene expression changes in 317 human iPSC lines derived from 101 donors and suggested that ∼50% of genome‐wide expression variations came from donor individuals.^12^ In light of the recent advance of understanding on the variant‐to‐function issue, that is, the causative role of genetic variants in diseases or phenotypic traits,^13^, ^14^ it would be highly speculable that a large proportion of variable iPSC phenotypes are likely a result of particular genetic variations.

## 3 DISEASE‐ASSOCIATED VARIATIONS ORIGINATING IN DONORS MAY CONSTRAIN THE APPLICATION OF MSCs

MSC heterogeneity exists among individuals, ages, tissue sources, cultural and cryopreservative conditions, passages, and even inter‐ or intracell colonies, resulting in their varied functional ability in vivo, which may affect the MSC use in regenerative medicine and their therapeutic efficacy.^6^, ^7^, ^8^, ^9^, ^15^ Interestingly, varied ex vivo growth kinetics of MSCs from different donors may also be an important affecting factor for processing and manufacturing cellular products.^16^, ^17^ A latest study using global transcriptomic analysis revealed the genetic role of glutathione S‐transferase theta 1 (GSTT1) gene present/null variations in the growth‐capacity of human MSC and GSTT1 was proposed as a novel genomic DNA biomarker for human MSC scalability.^17^ In the meanwhile, tremendous efforts have been made to reduce MSC heterogeneity and select certain “pure” or functional subpopulations of MSCs, by using clone selection, cell surface markers, single cell analysis, and other technologies.^18^, ^19^, ^20^, ^21^ On top of these, optimization and selection of donors by a broad genetic analysis may at least in part benefit therapeutic applications in which selected genetic factors can serve as general indicators of successful stem cell therapy.

Bone, cartilage, and joint surrounding tissues all have a mesenchymal origin. Incidentally, MSCs appear to be the most mature and the widely used stem cell for clinical therapies and research related to bone and cartilage diseases. Among them is osteoarthritis (OA), the most common and severe degenerative disease of the joints.^22^ MSC transplantation has successfully been used to treat OA in several animal models and has emerged as one of the most promising methods for OA therapy.^23^, ^24^ In addition, more than 50 proof‐of‐concept clinical trials using MSCs are registered on www.ClinicalTrials.gov. Many trials involve the intra‐articular injection of autologous bone marrow‐ or adipose‐derived MSCs. However, some studies showed that proliferation, chondrogenic differentiation, and adipogenic differentiation capacity of MSCs isolated from OA patients were significantly decreased.^25^ These results raise the possibility that the genetic background of OA patient‐derived MSCs may impair their therapeutic efficacy. The genetic architecture of OA is complex and may involve hundreds of genes. *Growth differentiation factor 5* (*GDF5*), which is thought to be indispensable for cartilage development and homeostasis, is a susceptibility gene.^26^, ^27^, ^28^ Mutations in *GDF5* can lead to severely impaired joints and skeletons in both mice and humans. Single‐nucleotide polymorphism (SNP) (rs144383) (+104T/C), which lies in the 5′ untranslated promoter region of GDF5, is strongly associated with OA and such a strong correlation between rs143383 and OA exists in multiple ethnic groups.^26^ The T allele of rs143383 is more likely to result in a 27% reduction of *GDF5* expression relative to C allele.^29^ About 80% of patients with OA carry the T allele, while the C allele exerts a lower risk of OA (30‐40%). Therefore, a minor but persistent imbalance of GDF5 expression throughout life may render an individual more susceptible to OA.

MSCs originating either from the bone marrow or residing in cartilage tissue are responsible for the homeostasis and regeneration of the cartilage. One may speculate that the precise balance of *GDF5* is a key factor affecting the physiological function of MSCs in cartilage and that rs143383 might be used as a selection parameter for MSC donors. This hypothesis should be tested to determine its effect of on autologous or allogeneic MSC proliferation, chondrogenic differentiation, and repair efficacy. If validated, it would be reasonable to recommend that rs143383 should be accounted for when identifying MSC donors for OA patients. This idea could theoretically be extended to any other SNPs already experimentally proven to exert an adverse/advantageous effect on MSCs.

Many degenerative or metabolic diseases are classified as complex disease with multiple genetic components,^30^, ^31^ such as type 2 diabetes and obesity and neurodegenerative diseases, and have proven to be perfect candidate diseases of stem cell treatment.^32^, ^33^, ^34^, ^35^, ^36^ From multiple GWAS projects for years, tens to hundreds of genetic variants have been suggested to possibly attribute to each of these diseases.^37^, ^38^, ^39^, ^40^ SNPs associated with or adjacent to those key genes with a proven or putative function in regulating specific cell types are particularly attracting intense attention. Such examples include rs7903146 (the TCF7L2 gene) with type 2 diabetes,^40^ rs356219 (the SNCA gene) with Parkinson's disease,^35^, ^37^, ^39^ and SNP rs2075650 (the TOMM40 gene) and rs4420638 (the APOE gene) with Alzheimer's disease.^38^ Therefore, considering the importance of SNPs in the etiology of many complex and multifactorial diseases, it is reasonable for us to recommend that stem cells carrying known disease‐causing or susceptibility SNPs should not be transplanted into recipients to treat the same disease. Such a recommendation of course does not mean to neglect many other sources of MSC heterogeneity and the top task would still be the optimization of stem cell dose, the route of cell administration, and the selection of most suitable disease stages, in order to obtain the optimal clinical outcomes.

## 4 CRUCIAL ROLES OF SNP‐ASSOCIATED HSC HETEROGENEITY IN CLINICAL OUTCOMES

Hematopoietic stem cell transplantation (HSCT), either allogeneic or autologous, remains the most commonly applied clinical stem cell‐based therapy that is broadly used to treat and even cure leukemias and other disorders of the blood and immune system.^41^, ^42^ In most cases, allogeneic transplantation may be the only available choice. The outcome of HSCT strongly depends on the quality of the human leukocyte antigen (HLA) match between the donor and recipient identified by high‐resolution HLA genotype and match techniques. Transplantation with suboptimally matched donor stem cells exerts an unacceptably high risk of immune rejection and other life‐threatening complications, such as disease relapse, graft vs host disease, and viral infection.

Despite stringent procedures to secure the best HLA matching, life‐threatening complications continue to occur following HSCT. Several studies have found that SNPs in genes related to innate and adaptive immune responses, residual leukemia, allo‐antigens, and drug metabolism are associated with HSCT outcomes and should be applied to identify patients at risk for post‐HSCT complications.^43^, ^44^, ^45^ A recent cohort analysis of 1157 HLA‐matched cases found that patients with donors homozygous for the C variant of rs10912564 in the key costimulatory molecule TNFSF4 had better disease‐free survival, overall survival with less treatment‐related mortality.^46^ The study further demonstrated that the TNFSF4C variant had a higher affinity for the transcription factor Myb and increased percentage of TNFSF4‐positive B cells compared with CT or TT genotypes, suggesting that the TNFSF4C variant may associate with outcomes. The CCR5 coreceptor on CD4^+^ T cells is critical for R5‐tropic HIV viral entry. In 2009, the “Berlin patient” presented with relapsed acute myelogenous leukemia and human immunodeficiency virus (HIV‐1) symptoms and underwent allogeneic HSCT derived from a donor lacking the CCR5 coreceptor (homozygous for the Δ32 deletion).^47^ Since then, HIV in the patient's blood and tissues has remained undetectable and antiretroviral therapy has been halted. Ten years later, a second individual (the “London patient”) with HIV‐1 infection and concomitant leukemia was cured by allogeneic HSCT with stem cells bearing the *CCR5*Δ32/Δ32 mutation.^48^ With the aim of mimicing these results while avoiding ethics and safety issues, the CRISPR/Cas9 gene editing technology was recently adopted to edit CCR5 in HSCs.^49^ The approach is expected to benefit more HIV patients and produce more “Berlin patients” and “London patients” in the future.

## 5 CONCLUSIONS

Stem cell and genomics research are two tightly connected areas, both of which advance rapidly and can provide perfect opportunities and examples for precision and personalized therapies. New technologies and facilities are now readily accessible in hospitals and laboratories and enable the goal. These include the availability of multiple stem cell sources, extensive next‐generation sequencing, as well as efficient and safe gene modification techniques.

Broad or even whole genome sequencing‐based gene testing has nowadays become acceptable more than ever before.^50^ Knowing the genetic traits may not only be benefit recipients but also a long‐term interest for donors. This may however be a dilemma considering that there may be potential ethical issues regarding the disclosure of personal information and that additional procedures are taken when screening a particular genetic background of donors. In addition, for most species and cell types, the amount of genomics data keeps accumulating daily and has reached multi‐Gigabyte levels. It would be ideal to assign functional significance to each genetic variation identified in a clinical context for a specific tissue or cell type. This requires functional assays in animal or stem cell models in addition to population‐level replication studies in designated clinical settings. As multiple SNPs are implicated in most clinical conditions, it would be necessary to establish a high‐throughput screening assay to assess the functional significance of a large number of genetic variants. For these purposes, CRISPR/Cas9 editing in iPSCs may serve as a good experimental model system to define the contribution of a gene or its variant to cell differentiation and regeneration.

Ultimately, any formal recommendations regarding the impact of SNPs carried by donor stem cells on the outcomes of stem cell therapy will require investigation in patients in the context of clinical trials, as performed for HSCT. Furthermore, we should acknowledge that attributing interdonor variations solely to genetics is difficult, given that different donors will have confounding variables such as unmatched lifestyle choices and experiences. Critics may argue that it is hard enough to find a match based on HLA, let alone including additional genetic criteria. Research is just beginning to address these questions and we think that the benefits to improved therapeutic outcome following stem‐cell transplantation will far outweigh these concerns.

## CONFLICT OF INTEREST

The authors declared no potential conflicts of interest.

## AUTHOR CONTIBUTIONS

T.W., J.Z.: conception and design, manuscript writing; J.Q.L.: reference selection and manuscript drafting; G.Q.Z., F.Z.: conception and design, manuscript writing, financial support, and final approval of the manuscript.
